# Mutant enzymes challenge all assumptions

**DOI:** 10.7554/eLife.02171

**Published:** 2014-02-11

**Authors:** Ryan M Nottingham, Suzanne R Pfeffer

**Affiliations:** 1 **Ryan M Nottingham** is in the Institute for Cellular and Molecular Biology, Department of Molecular Biosciences, The University of Texas at Austin, Austin, United Statesryan.nottingham@utexas.edu; 2 **Suzanne R Pfeffer** is an *eLife* reviewing editor, and is in the Department of Biochemistry, Stanford University School of Medicine, Stanford, United Statespfeffer@stanford.edu

**Keywords:** Membrane traffic, Rab GTPase, nucleotide exchange factor, Human

## Abstract

Enzymes called Rab GTPases that carry so-called “activating” mutations may never become activated at all.

**Related research article** Langemeyer L, Bastos RN, Cai Y, Itzen A, Reinisch KM, Barr FA. 2014. Diversity and plasticity in Rab GTPase nucleotide release mechanism has consequences for Rab activation and inactivation *eLife* **3**:e01623. doi: 10.7554/eLife.01623

**Image** Model showing the interaction between a Rab enzyme and guanine nucleotide exchange factor

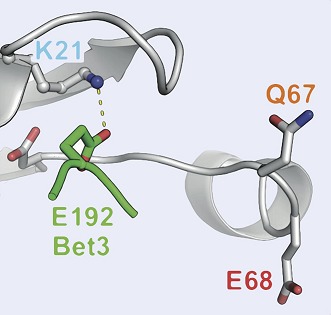

Human Rab GTPases are a family of about 60 enzymes that regulate a variety of processes within cells (Stenmark, 2009). Rab GTPases interconvert between an active state (in which a GTP molecule is bound to the enzyme) and an inactive state (in which a GDP molecule is bound to it; Barr and Lambright, 2010). Rab GTPases have been studied for several decades but now, in *eLife*, Francis Barr of Oxford University and co-workers—including Lars Langemeyer as first author—report the results of experiments that turn our assumptions about these enzymes upside down (Langemeyer et al., 2014).

Rab GTPases are activated by proteins called guanine nucleotide exchange factors, and they are de-activated by GAP proteins. During activation the exchange factors stimulate the release of a bound GDP molecule from the Rab enzyme, thus permitting a GTP molecule to bind it. De-activation involves the GAP proteins helping the Rab GTPase to hydrolyse a bound GTP molecule, which leaves a GDP molecule bound to the Rab.

Rab GTPases are part of a larger family of enzymes called the Ras-related proteins. GAP proteins catalyse the inactivation of Ras by inserting an arginine ‘finger’ into the nucleotide binding site of the protein (Scheffzek et al., 1997). A glutamine residue in the Ras protein helps to properly orient an active site water molecule that drives the hydrolysis of GTP to GDP. This glutamine is part of the conserved ‘switch II’ motif found in nearly all Ras-related proteins.

In 2006, however, researchers at UMass Medical School showed that Rab GAP proteins are not like Ras GAP proteins: a Rab GAP protein called Gyp1p employs a ‘dual-finger’ mechanism that uses both arginine and glutamine residues to de-activate a particular Rab GTPase called Rab33 (Pan et al., 2006). Unlike what happens with Ras, the glutamine residue in the switch II motif in Rab33 does not contribute directly to the catalysis of GTP hydrolysis: instead it is involved in the interaction between the Rab33 enzyme and the GAP protein. Moreover, mutation of the Rab switch II glutamine residue leads to only a 3–10 fold decrease in catalytic efficiency, whereas mutation of the arginine and glutamine fingers (which belong to the GAP protein) leads to a 100–1000 fold decrease (Pan et al., 2006). Similar findings were reported for a GAP protein called TBC1D20 that deactivates Rab1 (Figure 1A).

**Figure 1.**
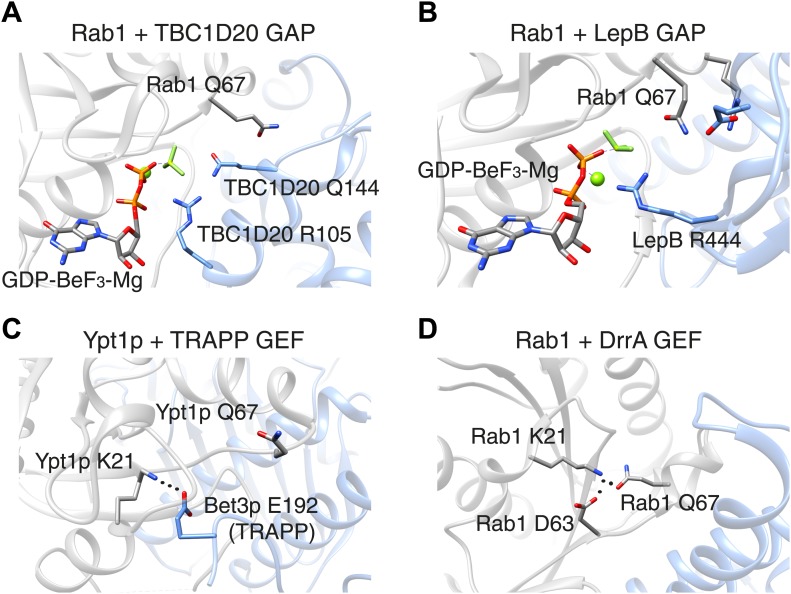
The switch II glutamine residue plays diverse roles in Rab1. (**A**) When the Rab1b enzyme (shown in grey) interacts with the GAP protein TBC1D20 (light blue; PDB 4HLQ), the switch II glutamine residue (Q67) is oriented away from the active site (which is at the centre of the figure). This means that it is not directly involved in the GAP-catalysed hydrolysis of GTP, but is still required for the interaction between the enzyme and the GAP protein (Gavriljuk et al., 2012). (**B**) When Rab1b interacts with the GAP protein LepB (PDB: 4I1O), the switch II glutamine residue is oriented toward the active site by residues that belong to the enzyme and the GAP protein (Gazdag et al., 2013; Mishra et al., 2013). (**C**) When the Ypt1p enzyme (which is the yeast equivalent of Rab1b) interacts with the guanine nucleotide exchange factor (GEF) called TRAPP (PDB: 3CUE), the switch II glutamine residue is oriented away from the nucleotide binding site of the enzyme, which is near a lysine residue (K21). This residue is stabilized by an interaction with a glutamic acid residue (E192) on the Bet3p subunit of the exchange factor, leading to the displacement of GDP from the nucleotide binding site. (**D**) When the Rab1b enzyme interacts with the exchange factor DrrA (PDB: 3JZA), the switch II glutamine residue is oriented toward the lysine residue. The interaction between these glutamine and lysine residues is further stabilized by interaction with an aspartate residue (D63) on Rab1b. GAP: GTPase-activating protein.

In Ras and other well studied Ras-related proteins, mutations of the switch II glutamine residues are classical activating mutations that block the intrinsic ability of these enzymes to hydrolyse bound GTP. Thus, a glutamine to leucine mutation is widely assumed to generate the active conformation of these proteins. However, for Rab GTPases, if the catalytic glutamine residue is supplied by the GAP protein, one cannot assume that such a mutation will be of any significant consequence in cells, since a GAP protein may still be able to inactivate this enzyme. Indeed, the yeast equivalent of Rab1 can be inactivated by a GAP protein, even when it harbours a mutation of the switch II glutamine residue (to leucine), both in vitro and in cells (De Antoni et al., 2002).

In these cases, it appears that the mutation of the switch II glutamine influences the interaction between the Rab GTPase and the GAP protein more than it influences the absolute hydrolysis capacity of the Rab enzyme. However, this is not true for all GAP proteins. Pathogenic bacteria often have virulence factors that mimic the functions normally performed by host cell proteins. The LepB protein from pathogenic *Legionella* is a Rab1 GAP protein that uses a distinct mechanism to stabilize the switch II glutamine residue in Rab1 in a catalytically competent conformation (Figure 1B). A mutation of the glutamine residue in this context causes a ∼10,000-fold decrease in catalytic efficiency (Gazdag et al., 2013; Mishra et al., 2013). This contrasts with GAP proteins from *Shigella* and certain forms of *E. coli* that catalyse the hydrolysis of the bound GTP molecule using the dual-finger mechanism, despite having structures that are very different from those of the GAP proteins found in the host cell (Dong et al., 2012).

Now Barr and co-workers—who are based at Oxford, Yale and the Technical University of Munich—have studied the impact of mutation of the glutamine residue in the switch II motif in three Rab GTPases: Rab1, Rab5 and Rab35. It used to be assumed that this mutation would result in active Rab enzymes. In Rab1 and Rab35, however, this mutation yields a form of the protein that cannot be activated by the relevant guanine nucleotide exchange factor: however, a different exchange factor was able to activate the mutant form of Rab1 (Figure 1C,D; Langemeyer et al., 2014). Moreover, while the mutant forms of Rab1 and Rab35 were poor substrates for certain GAP proteins, a GAP protein called RUTBC3 could bind to and act upon mutant Rab5 protein, similar to previous reports from other groups (see, e.g., Pan et al., 2006). The glutamine mutation had little effect on the intrinsic rate of GTP hydrolysis by Rab1, but a significant effect for Rab5. Thus, the switch II glutamine residues display much more diverse roles than previously thought.

Importantly, Langemeyer et al. show that rather than being ‘constitutively active’, and stimulating the trafficking of the Shiga toxin to the Golgi within cells, mutant Rab35 does not support this process under conditions where the wild type protein is fully competent. These findings indicate that the entire field of Rab biology has relied upon false assumptions. Mutant proteins that we thought could not be inactivated may, in fact, never have been activated in the first place, and residues that we presumed were important actually play roles quite unlike what we anticipated. This could have led to, for example, screens for Rab GTPase function completely missing important roles performed by these enzymes. Studies of host–pathogen interactions would also have been undermined: for example, while *Legionella* uses GAP proteins that rely on the switch II glutamine residue for their catalytic activity, *Shigella* does not.

As workers in this field attempt to clarify the roles of all 60 or so Rab GTPases, we must use great care when analysing and interpreting the behaviour of mutant Rab proteins. Rab GTPases can be activated and inactivated by a variety of molecular mechanisms, and even a single Rab GTPase can be modulated by multiple means. The large number of Rab GTPases (and related GAP proteins, exchange factors and other proteins) will each require careful analysis to understand their precise roles and the mechanisms they use to perform these roles in cells. Scientists beware: not all Rab GTPase switch II glutamine mutations represent constitutively active forms.
